# Differential MicroRNA Landscape Triggered by Estrogens in Cancer Associated Fibroblasts (CAFs) of Primary and Metastatic Breast Tumors

**DOI:** 10.3390/cancers11030412

**Published:** 2019-03-23

**Authors:** Adele Vivacqua, Maria Grazia Muoio, Anna Maria Miglietta, Marcello Maggiolini

**Affiliations:** 1 Department of Pharmacy, Health and Nutritional Sciences, University of Calabria, 87036 Rende (CS), Italy; mariagraziamuoio@libero.it; 2 Breast Unit, Regional Hospital Cosenza, 87100 Cosenza, Italy; annamariamiglietta@virgilio.it

**Keywords:** breast cancer, metastasis, CAFs, estrogens, microRNAs

## Abstract

Cancer associated fibroblasts (CAFs) play a main role in breast cancer progression and metastasis. Estrogens modulate in breast CAFs the expression of microRNAs (miRNAs) that are involved in the development of many tumors. In order to provide novel insights on the regulation of miRNAs by estrogens in breast cancer, we analyzed the expression of 754 miRNAs in CAFs obtained from primary mammary tumors and CAFs derived from a cutaneous breast cancer metastasis. Using the TaqMan™ Human MicroRNA Array, we found that 17β-estradiol (E2) modulates numerous peculiar and common miRNAs in CAFs derived from primary and the metastatic malignancies. In particular, we assessed that E2 modulates 133 miRNAs (41 up and 92 downregulated) in CAFs derived from primary breast tumors, whereas E2 modulates 415 miRNAs (399 up and 16 downregulated) in CAFs derived from a cutaneous metastasis of breast carcinoma. Therefore, a number of miRNAs three times higher in metastatic CAFs with respect to primary breast CAFs was found modulated by E2. Our findings shed new light on the cumulative regulation of miRNAs by E2 in the main players of the tumor microenvironment as CAFs. Moreover, our data may be taken into consideration that is useful toward innovative prognostic and therapeutic approaches in breast cancer progression.

## 1. Introduction

Breast cancer is the most common type of neoplasia and the primary cause of cancer mortality in women [1]. Deaths from breast tumors are mainly due to the metastatic dissemination of the malignant cells, a process that may occur in approximately 30% of patients [1]. Metastasis starts with the local invasion of the surrounding host tissues by cells originating from the primary tumor and then the malignant cells migrate to distant organs via either the blood stream or the lymphatic vessels [2]. It has been established that the interactions between breast cancer cells and the associated stroma play a crucial role in breast tumor progression and metastasis [3]. Within the microenvironment, the fibroblasts initially contribute to block the tumor growth. Thereafter, the fibroblasts change the morphology and function resulting in cancer associated fibroblasts (CAFs) upon the stimulation of various molecular signals generated by nearby tumorigenic cells [4,5]. CAFs are highly heterogeneous and express specific markers, which are widely used for their identification [6]. Previous studies have shown that CAFs are involved in all stages of tumorigenesis and their action may be associated with a poor survival outcome [6]. In this regard, it has been shown that secreting several factors, including interleukins, growth factors and collagen, CAFs may trigger the inflammatory microenvironment through either paracrine or autocrine mechanisms [4,7,8,9]. Within the neoplastic stroma, CAFs interact not only with the cancer cells but also with pro-tumoral cells like macrophages, regulatory T cells, myeloid-derived suppressor cells, cancer associated dendritic cells and others [6]. In addition, CAFs may negatively regulate certain functions of diverse cells like natural killer and cytotoxic T lymphocytes, which display an oncosuppressor activity in the tumor stroma [6].

Previous studies have demonstrated that estrogens contribute to the development of breast cancer mainly acting through the classical estrogen receptors (ERs) [10]; however, the G protein estrogen receptor (GPER) has been also involved in the estrogen stimulation of both ER-negative breast cancer cells and CAFs [11,12,13,14,15,16,17,18,19,20,21].

Recent evidence has indicated that estrogens may regulate in diverse cell types, including CAFs, the expression of microRNAs (miRNAs) [22,23,24,25] that are short molecules of non-coding RNA involved in important pathophysiological processes as tumor development [26,27]. In particular, miRNAs regulate gene expression binding to specific sequences located within the 3′ un-translated region (3′-UTR) of target mRNAs [28]. Different mechanisms as chromosomal abnormalities, epigenetic modifications and even an altered biogenesis and transcriptional control of miRNAs have been associated with their dysregulation in various types of cancer as breast tumors [29,30,31].

In order to provide novel findings on the potential of estrogens to modulate the expression of miRNAs in breast cancer microenviroment toward the metastatic dissemination, we aimed to assess upon exposure to estrogens the miRNA expression landscape in CAFs derived from primary breast tumors and CAFs obtained from a cutaneous metastasis of a mammary carcinoma. Intriguingly, we found that estrogens modulate the expression of miRNAs about three times more in metastasis derived CAFs with respect to CAFs obtained from primary breast tumors. Moreover, our data identified a variety of peculiar and common miRNAs regulated in the aforementioned cells, therefore providing a more comprehensive scenario on the potential of estrogens to engage a large number of miRNAs toward the dissemination of breast cancer cells.

## 2. Results and Discussion

**Figure 1:**
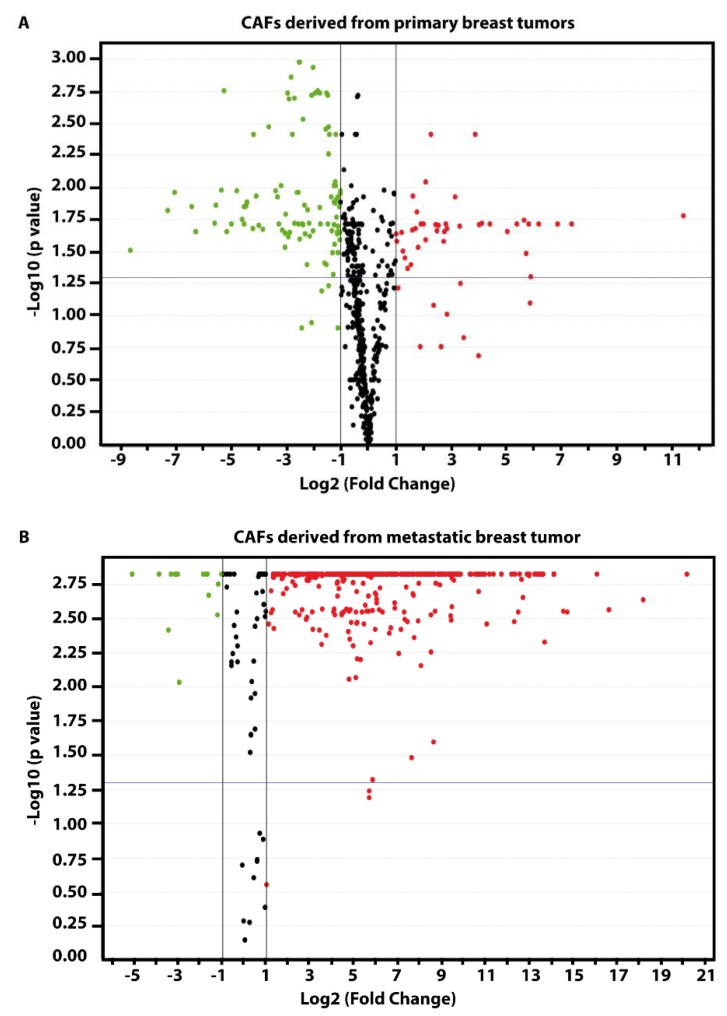
Volcano plot of miRNAs in cancer associated fibroblasts (CAFs) derived from primary breast tumors (**A**) and metastatic breast cancer (**B**) upon treatment with 100 nM E2 (17β-estradiol) for 4 h. The *x*-axis represents log2 Fold Change of miRNAs expression in estrogen stimulated samples versus vehicle treated samples, the *y*-axis represents –log10 *p*-values. The vertical dashed lines represent Fold Change = 2.0, while the horizontal dashed line represents *p*-value = 0.05. Points to the left (green) and right (red) of the plots represent miRNAs significantly downregulated and upregulated by E2 treatment, respectively.

**Figure 2:**
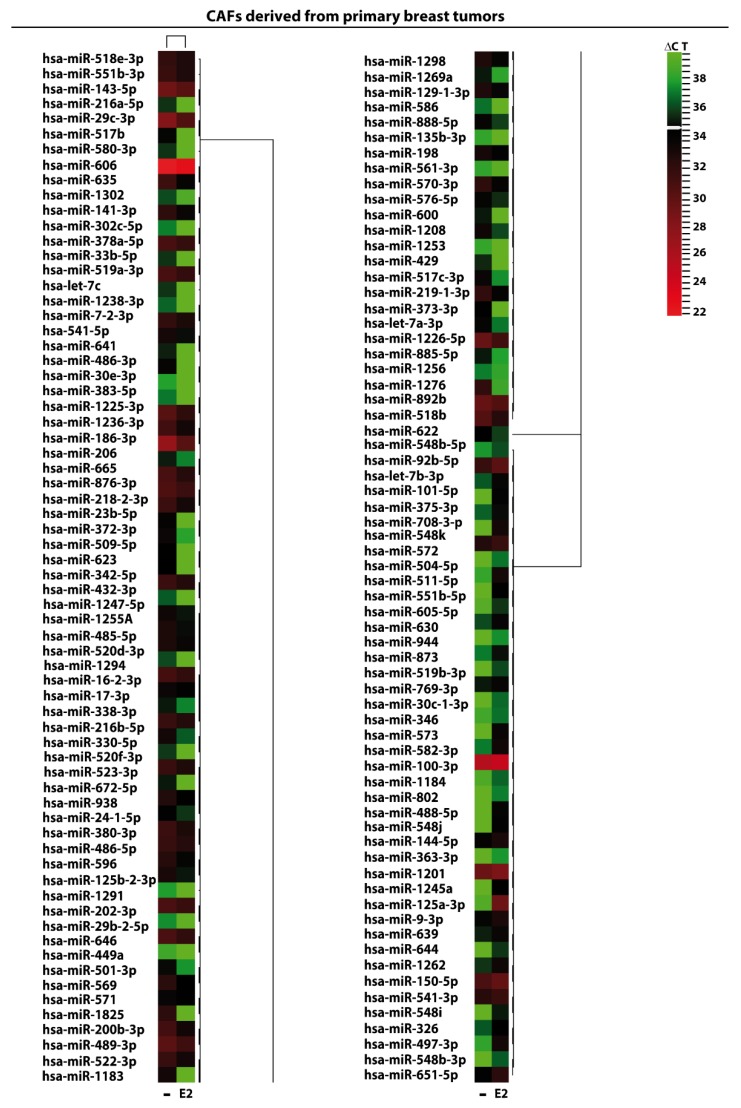
E2-modulated the expression of miRNAs in CAFs derived from primary breast tumors. Heat Map representation of 133 E2-regulated miRNAs in CAFs treated with 100 nM E2 for 4 h and analyzed by TaqMan Low-Density Array Human miRNA. The rows represent miRNA and columns represent the treatment exposure. Each column is illustrated according to a colour scale from green (low expression) to red (high expression). The distance measured is Euclidean Distance and the clustering method is complete linkage. Dendrograms of clustering analysis for miRNAs and samples are displayed on the top and right, respectively.

**Figure 3:**
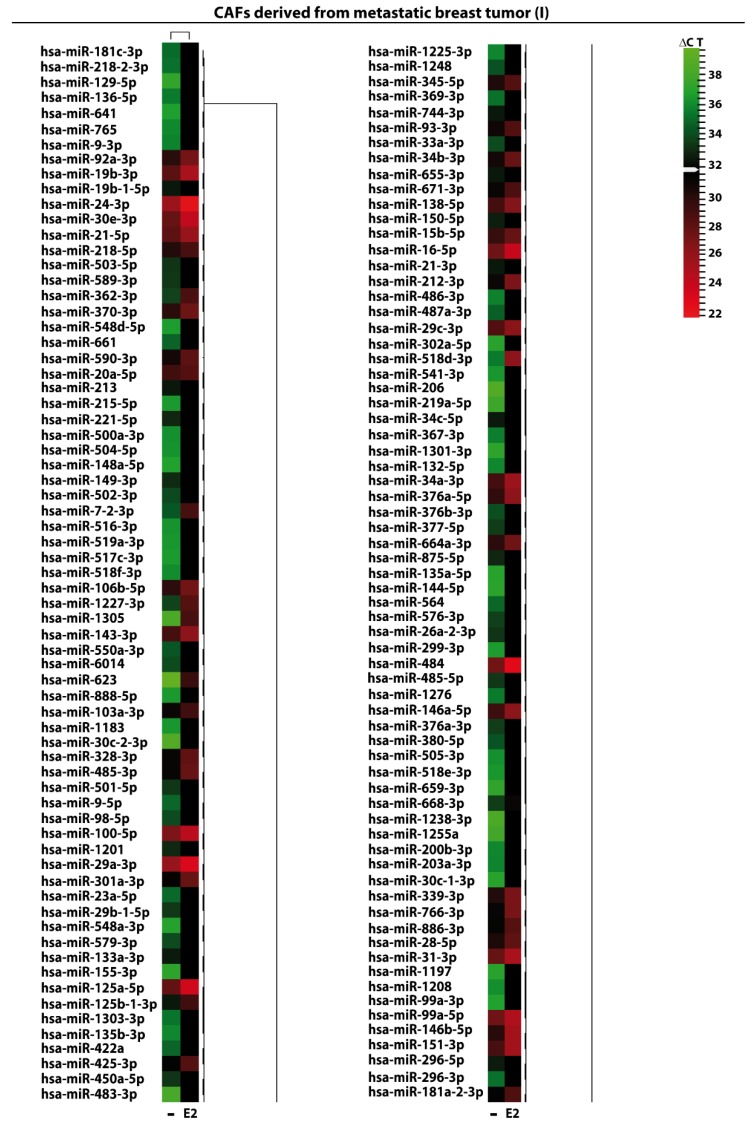
E2-modulated expression of miRNAs in CAFs derived from cutaneous metastatic breast tumor. Heat Map representation of 138 E2-regulated miRNAs in CAFs derived from a cutaneous metastasis treated with 100 nM E2 for 4 h and analyzed by TaqMan Low-Density Array Human miRNA. Rows represent an miRNA and columns represent the treatment exposure. Each column is illustrated according to a colour scale from green (low expression) to red (high expression). The distance measured is Euclidean Distance and the clustering method is complete linkage. Dendrograms of clustering analysis for miRNAs and samples are displayed on the top and right, respectively.

**Figure 4:**
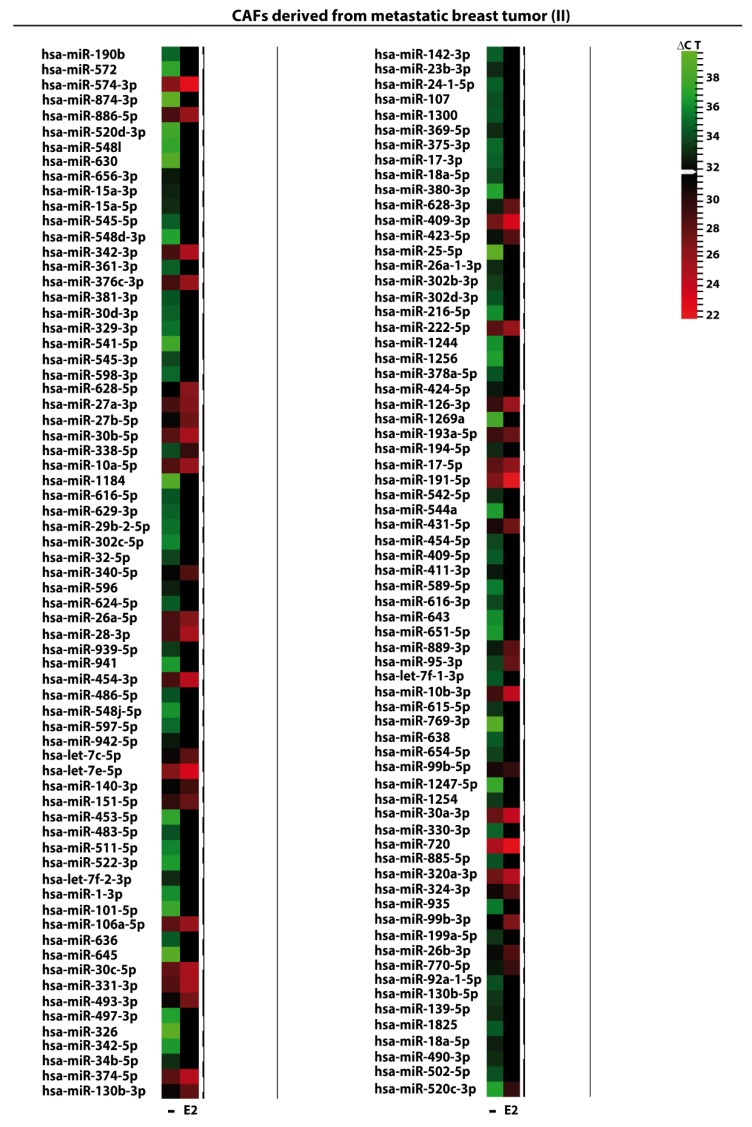
E2-modulated expression of miRNAs in CAFs derived from cutaneous metastatic breast tumor. Heat Map representation of 138 E2-regulated miRNAs in CAFs derived from a cutaneous metastasis treated with 100 nM E2 for 4 h and analyzed by TaqMan Low-Density Array Human miRNA. Rows represent an miRNA and columns represent the treatment exposure. Each column is illustrated according to a colour scale from green (low expression) to red (high expression). The distance measured is Euclidean Distance and the clustering method is complete linkage. Dendrograms of clustering analysis for miRNA and samples are displayed on the top and right, respectively.

**Figure 5:**
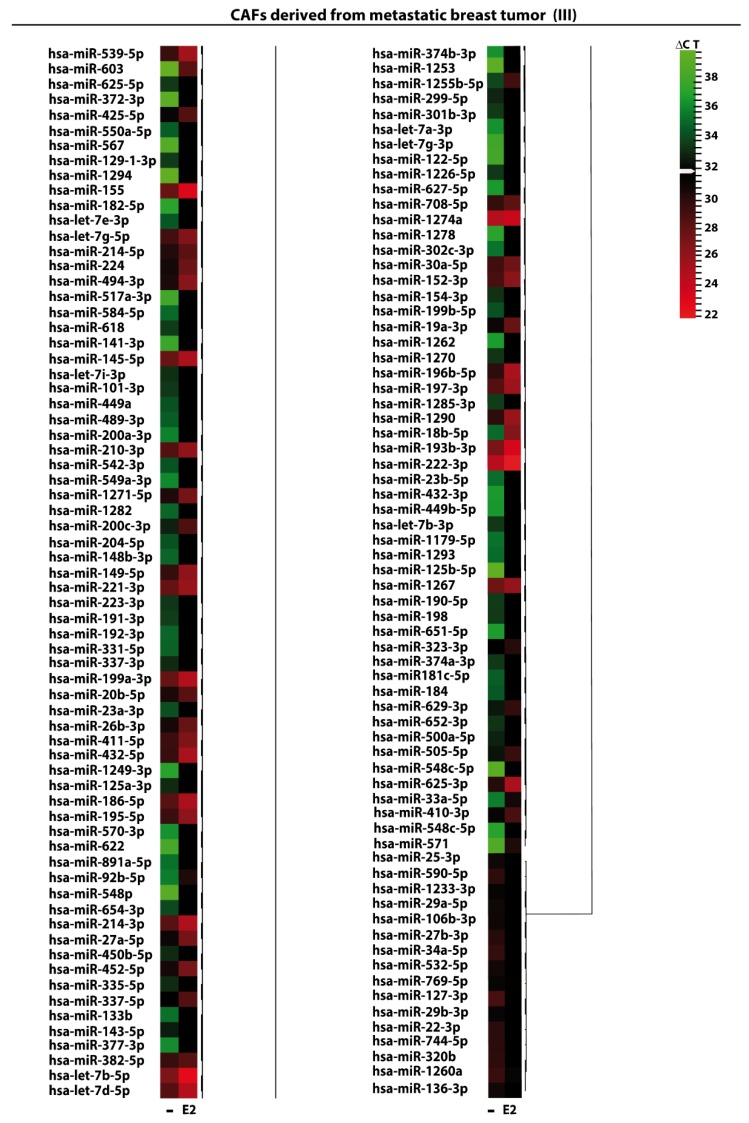
E2-modulated expression of miRNAs in CAFs derived from a cutaneous metastatic breast tumor. Heat Map representation of 139 E2-regulated miRNAs in CAFs derived from a cutaneous metastasis treated with 100 nM E2 for 4 h and analyzed by TaqMan Low-Density Array Human miRNA. Rows represent an miRNA and columns represent the treatment exposure. Each column is illustrated according to a colour scale from green (low expression) to red (high expression). The distance measured is Euclidean Distance and the clustering method is complete linkage. Dendrograms of clustering analysis for miRNAs and samples are displayed on the top and right, respectively.

**Figure 6:**
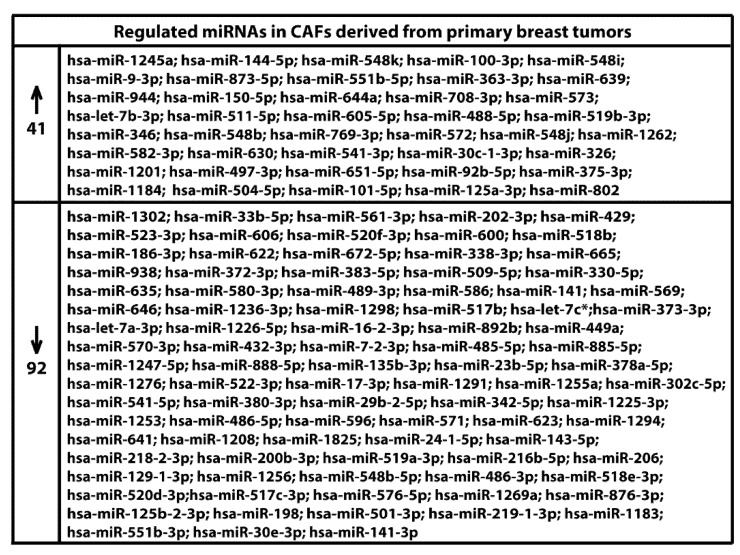
Identification of up (*n* = 41) and down (*n* = 92) regulated miRNAs in CAFs derived from primary breast tumors and treated for 4 h with 100 nM E2.

**Figure 7:**
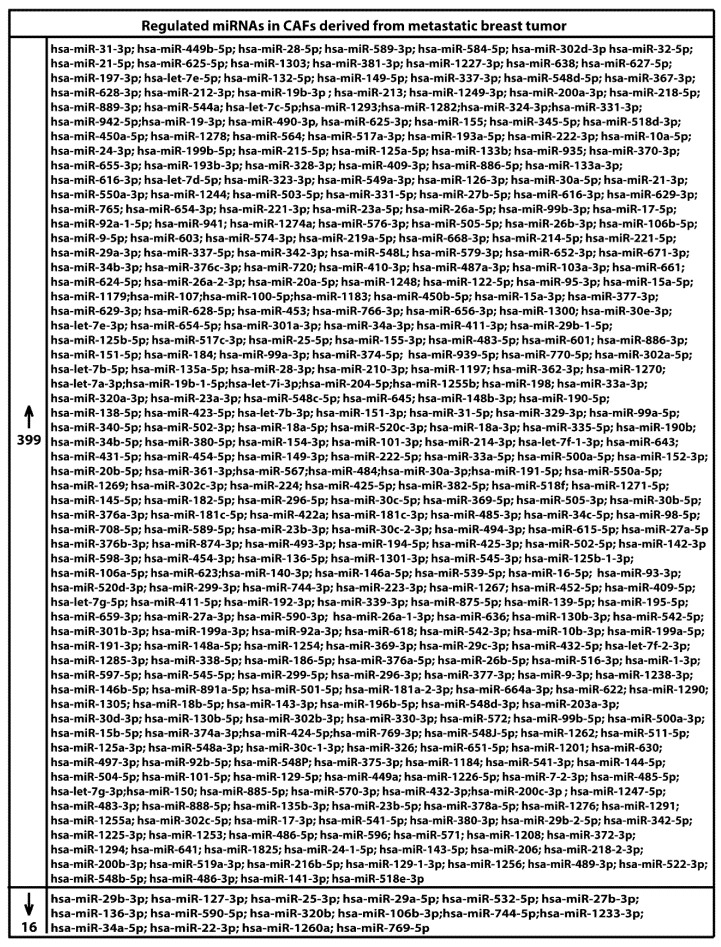
Identification of up (*n* = 399) and down (*n* = 16) regulated miRNAs in CAFs derived from a cutaneous metastasis of breast tumor and treated for 4 h with 100 nM E2.

**Figure 8:**
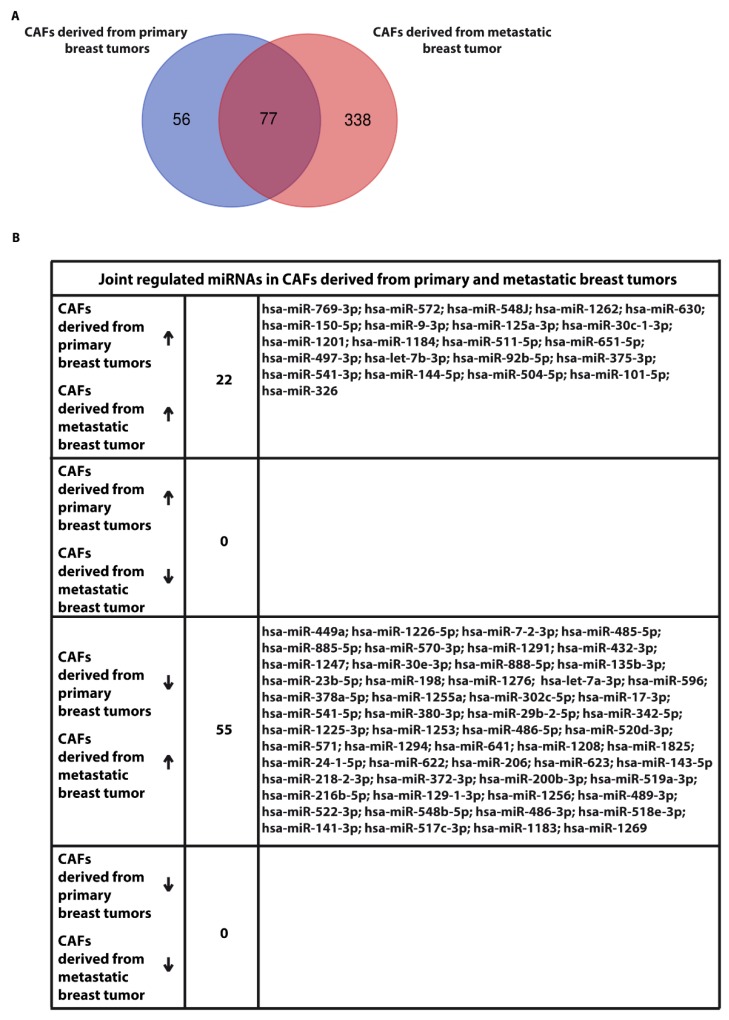
(**A**) Venn diagram of unique and joint E2-modulated miRNAs in CAFs derived from primary breast tumors and CAFs derived from a cutaneous metastasis of breast tumor. (**B**) up and downregulation of joint miRNAs (77) in CAFs derived from primary breast tumors and CAFs derived from a cutaneous metastasis of breast cancer and treated for 4 h with 100 nM E2.

**Figure 9:**
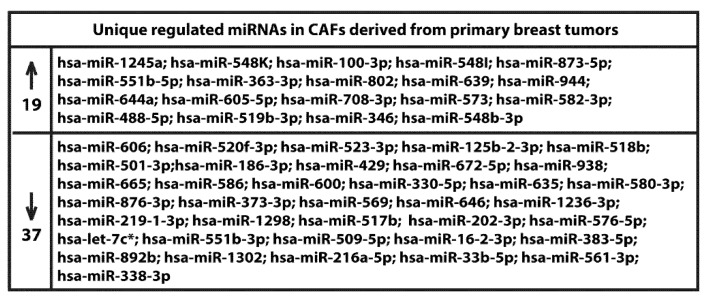
Up and downregulation of unique miRNAs in CAFs derived from primary breast tumors and treated for 4 h with 100 nM E2.

**Figure 10:**
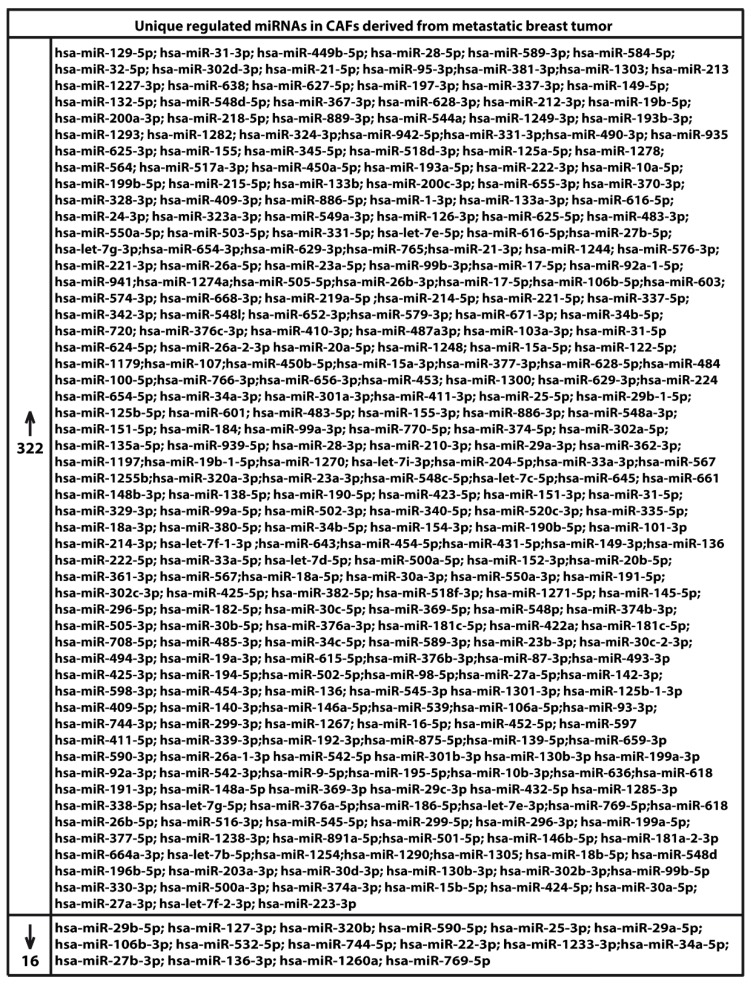
Up and downregulation of unique miRNAs in CAFs derived from a cutaneous metastasis of breast tumor and treated for 4 h with 100 nM E2.

**Figure 11:**
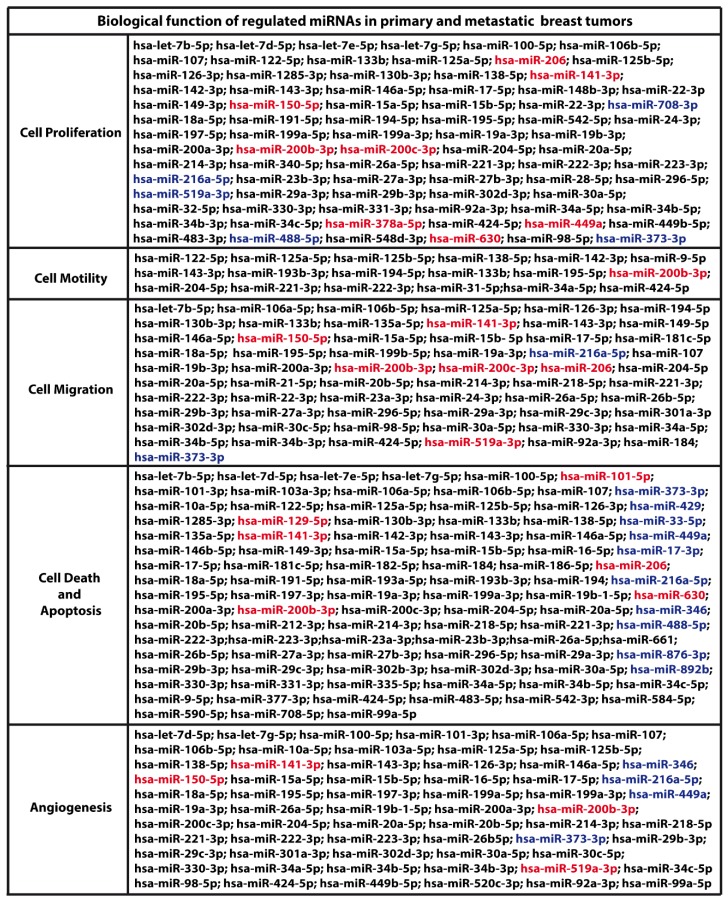
Biological function of miRNAs regulated by estrogens in CAFs derived from primary breast tumors (blue), metastatic breast tumor (black) or in both cell types (red). The analysis was performed by miRò v.2 on validated and predicted targets (number of tools predicting the interaction was at least 6).

We began the present study evaluating in CAFs derived from breast tumors and CAFs obtained from a cutaneous metastasis of a mammary carcinoma, the estrogen regulation of 754 miRNAs, which were formerly and recently involved in various human diseases, including cancer (www.thermofisher.com/order/catalog/product/4444913). The results obtained allowed us to define a more comprehensive profile of E2-modulated miRNAs in important components of the cancer microenvironment like CAFs located either within the primary tumor site or the distant metastasis. The aforementioned assessment was performed by the TaqMan™ Human MicroRNA Array (Applied Biosystems, Milan, Italy), setting the undetermined values at Ct 40. The levels of significance and the fold change in the expression of miRNAs in both cell types upon estrogen exposure were represented by a Volcano Plot (Figure 1A,B). Considering only the miRNAs exhibiting at least either a two-fold increase or a 50% reduction upon E2 treatment with respect to vehicle-treated cells and a statistical significance of *p* < 0.05, we identified 133 (Figure 2) and 415 (Figure 3, Figure 4 and Figure 5) E2-modulated miRNAs, respectively, in CAFs derived from primary breast tumors and CAFs derived from the metastatic breast cancer. These results demonstrate that E2 orchestrates the miRNA expression three times more in CAFs located within the metastatic breast cancer with respect to primary breast tumors. In particular, E2 upregulated 41 miRNAs and downregulated 92 miRNAs in CAFs derived from primary breast tumors (Figure 6), whereas the treatment with E2 increased the expression of 399 and lowered the expression of 16 miRNAs in CAFs derived from the metastatic breast cancer (Figure 7). Therefore, it could be argued that, in the latter type of cells, the estrogen stimulation triggers a huge miRNAs increase with respect to CAFs from primary breast tumors. Next, we aimed to assess the joint and unique regulated miRNAs in these cell types. As made evident by the Venn diagram, 56 and 338 unique E2-regulated miRNAs were detected, respectively, in CAFs derived from primary breast tumors and CAFs obtained from the metastatic breast cancer, whereas 77 miRNAs were found joint in these cell types (Figure 8A). Regarding the above-mentioned 77 miRNAs, upon E2 treatment 22 miRNAs increased in both cell types, whereas 55 miRNAs were found downregulated and upregulated, respectively, in CAFs derived from primary breast cancers and CAFs derived from the metastatic breast tumor (Figure 8B). Thus, within the joint miRNAs, only one third displayed a similar response, while the others showed an opposite regulation. In addition, E2 triggered an increase and a reduction, respectively, of 19 and 37 unique miRNAs in CAFs derived from primary breast tumors (Figure 9), whereas E2 upregulated 322 unique miRNAs and downregulated 16 unique miRNAs in CAFs derived from the metastatic breast cancer (Figure 10). Then, we performed an in silico analysis using MiRò v.2 software (microrna.osumc.edu/miro/) in order to ascertain relevant biological function of estrogen-modulated miRNAs as cell proliferation, motility, migration, apoptosis and angiogenesis (Figure 11).

Estrogens play a main role in the growth, development and function of the female reproductive system [32]. However, the action of estrogens is also involved in numerous diseases, including breast cancer [33]. In this regard, estrogens have been shown to trigger various signaling pathways through ER and GPER toward the stimulation of breast cancer cells and CAFs [11,34,35]. In addition, several studies have ascertained that estrogens regulate the expression of miRNAs in the aforementioned cell contexts [22,25,34,35,36,37]. In accordance with these data, our current results demonstrate that E2 stimulates the expression of numerous miRNAs in CAFs derived from primary breast tumors and CAFs obtained from a metastatic breast cancer. Considering that both cell types of CAFs are ER-negative but express GPER (see material and methods section), the modulation of miRNAs induced by E2 in the primary and the metastatic CAFs may involve GPER as ascertained in our and other investigations [22,25,38,39]; however, further studies are needed to better define the mechanisms involved in the regulation of miRNAs by estrogens.

The ability of cancer cells to metastasize is often related to certain properties deriving from molecular alterations, chemo-resistance and the activation of several transduction pathways [40]. In this context, previous studies have indicated that CAFs contribute to the metastatic process [41]. In particular, it has been made evident that, in primary sites of diverse types of tumor including breast cancer, CAFs stimulate the production of growth factors and cytokines, and promote the epithelial mesenchymal transition and other important biological outcomes, which trigger the growth and invasive features of near cancer cells [4,41,42]. Among other actions, CAFs also induce the mesenchymal to epithelial transition at the metastatic sites, hence facilitating the colonization of cancer cells [43]. Likewise, the involvement of certain miRNAs has been demonstrated in the metastatic process of breast tumors [44,45]. For instance, the metastasis was suppressed restoring the expression of miRNAs lost in experimental models of breast cancer ([44] and references therein). Indeed, miRNAs may act in different phases of the metastatic process including migration and invasion, epithelial mesenchymal transition, anoikis survival, intravasation and extravasation and then distant organ colonization [45,46]. Therefore, a better knowledge on the regulatory role exerted by miRNAs in the metastatic tumor progression would be useful toward new therapeutic approaches. In this regard, it should be mentioned that the role of miRNAs in a variety of cancer cells has been acknowledged [30], yet few data are available on the dysregulation of miRNAs in CAFs [47,48]. It is worth noting that the present investigation assessed that estrogens regulate numerous miRNAs involved in relevant biological responses that characterize tumor progression as cell proliferation, motility, migration, cell death, apoptosis and angiogenesis (Figure 11) [46,49]. In particular, we proved that estrogens trigger a higher amount of these miRNAs in CAFs derived from metastatic breast cancer with respect to CAFs derived from primary mammary tumors (Figure 11). Therefore, our results open new avenues toward a more comprehensive assessment on the role of certain miRNAs engaged by E2 in breast CAFs, in particular for the identification of specific miRNAs involved in the metastatic process. Our data also establish the basis of further studies aimed to assess the specific cellular responses involved by miRNAs in both primary and metastatic breast CAFs and to determine the molecular interactions of a single or a group of miRNAs prompting the metastasis of mammary tumors.

## 3. Materials and Methods

### 3.1. Reagents

17β-estradiol (E2) was purchased from Sigma-Aldrich Corp. (Milan, Italy) and solubilized in dimethyl sulfoxide (DMSO).

### 3.2. Cell Cultures

CAFs were obtained from mastectomy of invasive mammary ductal carcinoma of three patients and a cutaneous metastasis in a patient with the same type of tumor, who previously had undergone surgery. Briefly, samples were cut into smaller pieces (1–2 mm diameter), placed in digestion solution (400 IU collagenase I, 100 IU hyaluronidase, and 10% FBS, containing antibiotic and antimycotic solution) and incubated overnight at 37 °C. Cells were then separated by differential centrifugation at 90× *g* for 2 min. Supernatant containing fibroblasts was centrifuged at 485× *g* for 8 min; the pellet obtained was suspended in fibroblasts’ growth medium (Medium 199 and Ham’s F12 mixed 1:1 and supplemented with 10% FBS) and cultured at 37 °C in 5% CO_2_. CAFs were characterized by immunofluorescence. Cells were briefly incubated with human anti-vimentin (V9, sc-6260) and human anti-cytokeratin 14 (LL001, sc-53253), both from Santa Cruz Biotechnology (DBA, Milan, Italy) (Figure S1). To characterize fibroblasts’ activation, the anti-fibroblast activated protein α (FAPα) antibody was used (SS-13, sc-100528; Santa Cruz Biotechnology, DBA, Milan, Italy) (Figure S1). In both cell types, the expression of ERα (F-10, sc-8002; Santa Cruz Biotechnology, DBA, Milan, Italy) and GPER (AB137479) (Abcam, Euroclone, Milan, Italy) were analyzed by Western blotting [50]. Cells were grown in a 37 °C incubator with 5% CO_2_ in a mixture of Medium 199 and Ham’s F-12 (1:1) supplemented with 10% FBS and 100 μg/mL of penicillin/streptomycin (Gibco, Life Technologies, Milan, Italy). CAFs derived from the three invasive mammary ductal carcinoma were pooled into one sample to perform the following experimental assays.

### 3.3. Immunofluorescence Microscopy

Cells were grown in regular media on a cover slip and then fixed in ice-cold methanol at room temperature for 10 min, permeabilized with 0.2% Triton X-100, washed three times with PBS and incubated with 1% bovine serum albumin (BSA) in PBS at room temperature for 1 h. After washing with PBS, cells were incubated with primary antibodies against vimentin (V9), cytokeratin 14 (LL001) and FAPα (H-56) (Santa Cruz Biotechnology, DBA, Milan, Italy) (diluted in 1% BSA/PBS) at 4 °C for 18 h. After incubation, cells were washed three times with PBS and incubated with Alexa fluor conjugated secondary antibodies (Thermofisher Scientific, Milan, Italy) for 1 h at room temperature. Finally, cells were washed three times with PBS, incubated with DAPI (4′,6-diamidino-2-phenylindole) (1:1000) for 3 min and, after washing, immunofluorescence images for the characterization of CAFs were obtained by Cytation 3 Cell Imaging Multimode Reader and analyzed using the software Gen5 (BioTek, Ahsi, Milan, Italy). 

### 3.4. RNA Extraction

Cells were maintained in regular growth medium and then switched to medium lacking serum before the treatment with E2 100 nM for 4h. Total RNA was extracted from cultured cells using miRVana Isolation Kit (Ambion, Life Technologies, Milan, Italy) in accordance with the manufacturer’s recommendations. The RNA concentrations were determined using Gene5 2.01 Software in Synergy H1 Hybrid Multi-Mode Microplate Reader (BioTek, AHSI, Milan, Italy).

### 3.5. miRNA Expression Profiling

TaqMan™ Array Human MicroRNA A + B Cards Set v3.0 was used for global miRNA profiling. The panel includes two 384-well microfluidic cards (human miRNA pool A and pool B) that contain primers and probes for 754 different miRNAs in addition to small nucleolar RNAs that function as endogenous controls for data normalization. Equal quantity (100 ng) of RNA extracted from CAFs derived from primary breast tumors and a cutaneous metastasis upon treatment with vehicle or 100 nM E2 for 4 h was reverse-transcribed for cDNA synthesis using the Megaplex RT Primer Pool A or B and the TaqMan MicroRNA Reverse Transcription kit in a final volume of 7.5 μL (Applied Biosystems, Life Technologies, Milan, Italy). The reverse transcription reaction was incubated for 2 min at 16 °C, 1 min at 42 °C and 1 s at 50 °C for 40 cycles, followed by 5 min at 85 °C to deactivate the enzyme. The cDNA obtained was pre-amplified using Megaplex Preamp primer pool A or B and TaqMan PreAmp Master Mix 2× in a final volume of 25 μL using the same temperature conditions above described. The product was diluted 1:4 in Tris-EDTA (TE) 0.1×, to which were added TaqMan Universal Master Mix without Uracil-N Glycoslyase (UNG) 2× and nuclease free water. In addition, 100 μL of the sample/master mix for each multiplex pool were loaded into fill reservoirs on the microfluidic card. The array was then centrifuged, mechanically sealed with the Applied Biosystems sealer device and run on QuantStudio 6&7 Flex Real Time PCR System (Applied Biosystems, Life Technologies, Milan, Italy). Array experiments were performed in triplicate using samples from three independent RNA extractions.

### 3.6. Data Analysis

Raw array data were analysed by DataAssist^TM^ v3.01 (Applied Biosystems, Life Technologies, Milan, Italy) The baseline was set automatically, while the threshold was set manually at 0.2. Samples with Ct values undetermined were set to 40 and considered for the analysis. Each miRNA was normalized against the mean of the four RNU6B and its expression was then assessed in the E2 treated cells against the vehicle treated cells using the 2^−ΔΔCT^ method [51]. Differences between groups were calculated with the Student’s *t*-test. A volcano plot was obtained by DataAssist^TM^ v3.01 setting the Fold Change boundary = 2 and *p* value *p* = 0.05. miRNAs showing an increased value of two-fold expression and a 50% reduction respect to vehicle-treated cells and with *p* < 0.05 were selected. Venn diagram was obtained by an online bioinformatic tool [52]. Functional analysis of E2-modulated miRNAs was performed by the miRò v.2 software online (microRNA@osumc.edu).

### 3.7. Declarations: Ethics Approval and Consent to Participate

All procedures conformed to the Helsinki Declaration for the research on humans. Signed informed consent was obtained from the patients and the experimental research has been performed with the ethical approval provided by the “Comitato Etico Regione Calabria, Cosenza, Italy” (Approval Code: 166, 2 December 2016).

## 4. Conclusions

Breast cancer metastasis is an intricate process involving many factors and transduction pathways. Therefore, the identification of novel tools to detect and predict breast cancer metastasis at an early stage is important for the therapeutic management of this disease. In the last few years, emerging evidence has suggested the potential use of miRNAs as prognostic and therapeutic tools in breast cancer metastasis [45]. In this context, our results show a peculiar estrogen-modulated expression of miRNAs in metastatic CAFs with respect to CAFs of primary mammary tumors. Therefore, our data may open new avenues in order to assess further molecular targets useful in innovative prognostic and therapeutic approaches of breast cancer.

## Supplementary Materials

The following are available online at https://www.mdpi.com/2072-6694/11/3/412/s1, Figure S1: Representative images of CAFs derived from primary (A) and metastatic (B) breast tumors. CAFs were immunostained by anti-FAPα, anti–Vimentin and anti-Cytokeratin14 antibodies. Green signal: FAPα and Cytokeratin14; Red signal: Vimentin; Blue signal: Nuclei. Scale bar: 200 µm.
