# Supplementary material for: Differential MicroRNA Landscape Triggered by Estrogens in Cancer Associated Fibroblasts (CAFs) of Primary and Metastatic Breast Tumors
Source: Cancers (Basel). 2019 Mar 23;11(3):412. doi: 10.3390/cancers11030412 (PMC6468788; doi:10.3390/cancers11030412)
Supplement: Supplementary file 1 [file cancers-11-00412-s001.pdf]

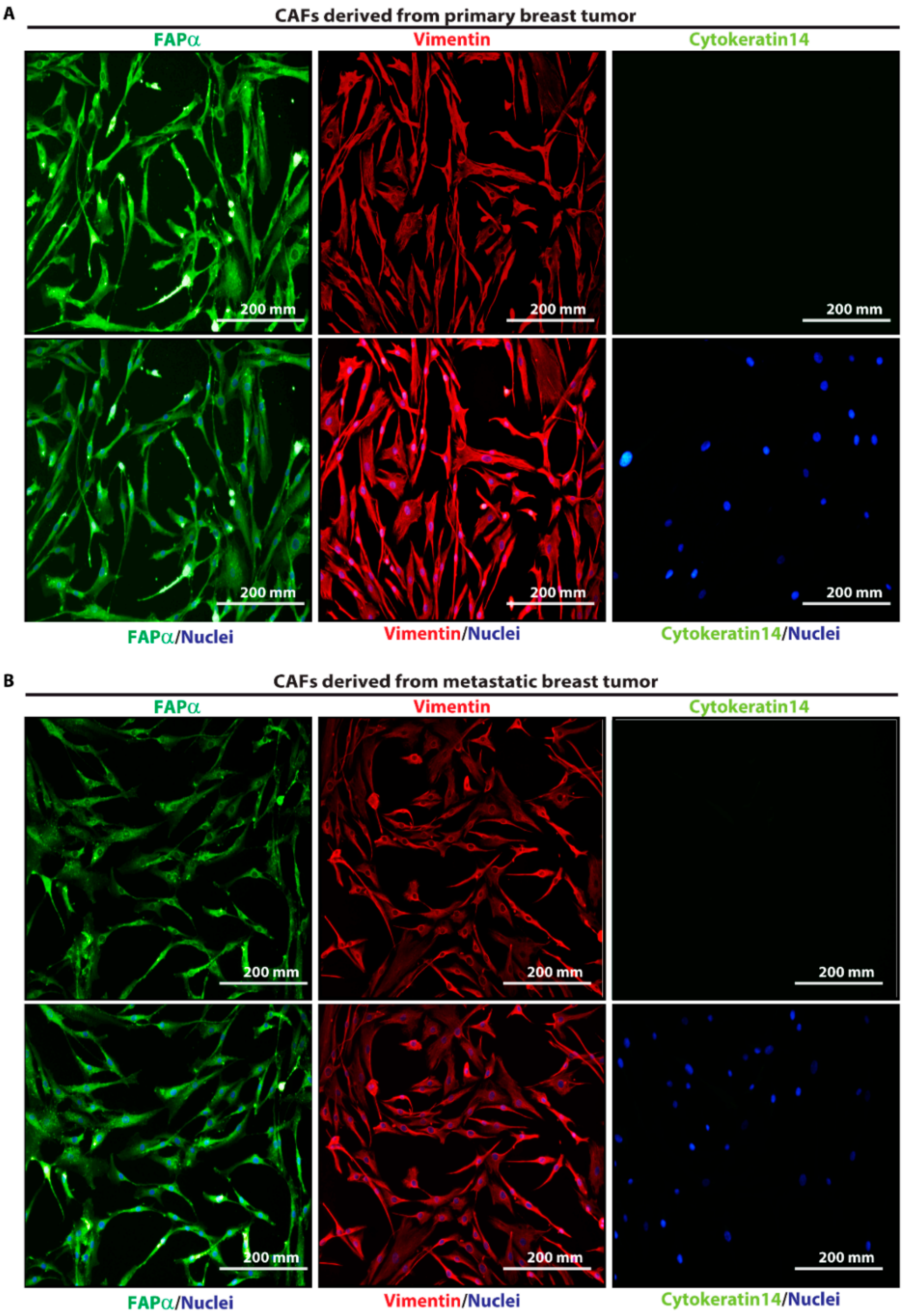

Figure S1. Representative images of CAFs derived from primary (**A**) and metastatic (**B**) breast tumors. CAFs were immunostained by anti-FAPα, anti–Vimentin and anti-Cytokeratin14 antibodies. Green signal: FAPα and Cytokeratin14; Red signal: Vimentin; Blue signal: Nuclei. Scale bar: 200μm.
